# Biochemical effects of mutations in the gene encoding the alpha subunit of eukaryotic initiation factor (eIF) 2B associated with Vanishing White Matter disease

**DOI:** 10.1186/s12881-015-0204-z

**Published:** 2015-08-19

**Authors:** Noel C. Wortham, Christopher G. Proud

**Affiliations:** Centre for Biological Sciences, University of Southampton, Life Sciences Building 85, Highfield Campus, Southampton, SO17 1BJ UK; South Australian Health and Medical Research Institute, PO Box 11060, SA5001 Adelaide, Australia

**Keywords:** CACH, Leukodystrophy, eIF2B, EIF2B1, VWM

## Abstract

**Background:**

Leukoencephalopathy with Vanishing White Matter (VWM) is an autosomal recessive disorder caused by germline mutations in the genes *EIF2B1-5*, which encode the 5 subunits of the eukaryotic translation initiation factor eIF2B. To date, analysis of the biochemical effects of mutations in the *EIF2B2-5* genes has been carried out, but no study has been performed on mutations in the *EIF2B1* gene. This gene encodes eIF2Bα, the smallest subunit in eIF2B which has an important role in both the structure and regulation of the eIF2B complex.

**Methods:**

eIF2B subunits were overexpressed in HEK293 cells and isolated from the resulting cell lysates by affinity chromatography. Formation of the eIF2B complex and binding of its substrate, eIF2, was assessed by western blot. Assays of the guanine nucleotide exchange (GEF) activity were also carried out.

**Results:**

Of the 5 eIF2Bα mutations studied, we found 3 that showed loss or reduction of binding of eIF2Bα to the rest of the complex, one with increased GEF activity, and one where no effects on activity or complex formation were observed.

**Conclusions:**

This is the first study on eIF2Bα VWM mutations. We show that some mutations cause expected decreases in GEF activity or complex formation, similar to a majority of observed VWM mutations. However, we also observe some unexpected changes which hint at other effects of these mutations on as yet undescribed functions of eIF2B.

## Background

Leukoencephalopathy with vanishing white matter (VWM; also known as childhood ataxia with central nervous system hypomyelination (CACH)) is an autosomal recessive neurological disorder with variable features including progressive cerebellar ataxia, spasticity and cognitive impairment [1]. VWM is characterised by chronic degradation of central nervous system white matter due to demyelination punctuated by episodes of rapid deterioration following insults including head trauma, infection with fever and acute fright [1].

Patients with VWM exhibit a broad range of disease severities, from mild adult-onset VWM, where patients exhibit less severe symptoms and survive many years post diagnosis, to the most severe congenital form of the disease, where patients are born with the disease and, at best, survive only a few months [2, 3]. The disease manifests in patients’ glial cells, particularly the astrocytes and oligodendrocytes, the latter being responsible for myelination in the central nervous system [4]. Studies in a mouse model of VWM have shown both immature myelination of white matter neurons, and a defect in the inflammatory response, mediated by astrocytes, in response to lipopolysacharride [5, 6].

VWM is caused by mutations in the *EIF2B1-5* genes encoding the subunits of the eukaryotic translation initiation factor eIF2B [3, 7]. eIF2B is a heterodecameric complex comprising 5 subunits, termed α through ε in order of increasing size, that acts as the guanine nucleotide exchange factor (GEF) for the G-protein eIF2 [8]. GTP-bound eIF2 is responsible for loading the initiator methionyl-tRNA onto the ribosome to allow initiation of protein synthesis to take place [9]. Hence, eIF2B is a key factor controlling the rate of protein synthesis in cells. eIF2Bε contains the catalytic domain towards its C-terminus [10]. The eIF2B subunits can be categorised based on sequence homology and complex formation in yeast [11]. eIF2Bγ and eIF2Bε form the catalytic subcomplex [12, 13]. eIF2Bα, β and δ form the regulatory subcomplex, since this complex confers regulation by increased phosphorylation of eIF2 which occurs in response to cellular stresses [14]. In particular, eIF2Bα has been shown to be required to mediate inhibition of eIF2B by phospho-eIF2 [15, 16]. In mammalian cells, three eIF2B complexes can be formed: eIF2Bα-ε form a decameric complex (actually eIF2B(αβγδε)_2_ with 100 % relative activity; eIF2B(βγδε) is a tetrameric complex with 50 % relative activity; and eIF2B(γε) is a dimeric complex with 20 % activity [2, 8]. The eIF2B holocomplex comprises two eIF2B(βγδε) heterotetramers held together by an eIF2Bα dimer, although eIF2Bα dimerisation is not required to link the heterotetramers [8].

One of the key regulatory mechanisms of eIF2B is mediated by phosphorylation of the α subunit of its substrate, eIF2, at Ser51 in response to cellular stresses including viral infection, amino acid deprivation and accumulation of unfolded proteins in the endoplasmic reticulum [17]. Phosphorylated eIF2 binds more strongly to, and inhibits, eIF2B, thereby preventing the recycling of non-phosphorylated eIF2. GDP [16]. This leads to inhibition of general mRNA translation, although some mRNAs are actually translated more efficiently, due to the presence of specific features in their 5′-untranslated regions, for example, the transcription factor ATF4 [18]. It has previously been shown that inappropriate activation of this pathway through inhibition of eIF2B by VWM-associated mutations leads to accumulation of ATF4 and its transcriptional targets [19–21].

To date, >150 individual VWM-associated mutations have been identified, some being present homozygously in particular patients, while others are compound heterozygous [3]. The majority of VWM mutations occur in the *EIF2B5* gene, which encodes the eIF2Bε subunit. A limited genotype-phenotype relationship has been established whereby the severity of the disease correlates with particular mutations [3]. For example, mutations in the *EIF2B4* gene, encoding eIF2Bδ, appear to result in more severe disease [2, 3]. The functional basis for this is, however, unknown. We and others have previously shown that some of these mutations result in changes in GEF activity and/or formation of the eIF2B complex [2, 22, 23].

The relationship of these changes to disease severity remains controversial. It has been suggested that defects in GEF activity are predictive of disease severity [24]. However, we have shown that disease severity seems unrelated to GEF activity defects, finding in some cases that mutations resulting in very severe disease have little or no defect in either complex formation or GEF activity [2]. This suggests that there are perhaps other functions of eIF2B that may also be affected. Indeed, Jennings *et al.* recently showed that eIF2B is required to dissociate eIF2. GDP from eIF5 (which possesses a GDP-dissociation inhibitor (GDI) activity [25]) in order to allow eIF2B to carry out GEF activity, thus giving it a function as a GDI-dissociation factor (GDF) [26]. Their studies in yeast established that this activity only requires eIF2Bγ and ε and showed that eIF2B complexes deficient in this GDF activity are able to slow translation in a similar manner to some VWM mutations [26]. Since GEF activity assays utilise purified eIF2, this activity is unlikely to have been measured in previous studies.

To date, six VWM-associated mutations have been identified in the EIF2B1 gene in patients (Table 1), including four missense mutations, one in-frame deletion and one nonsense mutation. While mutations in other subunits have been examined, no comprehensive study of mutations affecting eIF2Bα has been carried out. Unfortunately, information regarding disease severity of patients with these mutations is limited. The only patient with a described phenotype is one homozygous for Val183Phe, who exhibited the milder late juvenile/early adulthood disease [27]. eIF2Bα is unique among the eIF2B subunits in that, in yeast, it is the only subunit whose deletion can be tolerated [28]. Furthermore, it has been shown to have ‘moonlighting’ roles outside of the eIF2B complex, through interaction with the β-adrenergic receptor at the cell membrane [29]. We have previously shown that the Val183Phe variant disrupts formation of eIF2Bα homodimers, but does not affect formation of eIF2B decamers [8]. Furthermore, Richardson *et al.* [30] reported decreased stability of the Asn208Tyr variant in yeast cells. Here, we have examined the biochemical effects of VWM mutations in the *EIF2B1* gene, encoding the eIF2Bα subunit. We have studied both the ability of the mutated subunit to incorporate into eIF2B complexes, including binding of eIF2 and phospho-eIF2, and the effect of these mutations on GEF activity.

**Table 1:** VWM associated mutations in EIF2B1

DNA^a^	Protein^b^	Disease severity	Zygosity	Reference
IVS2+1G>A^c^	p.Ser84ins22aa, stop	Not reported	Heterozygous with Asn208Tyr	[7]
c.547G>T	p.Val183Phe	Juvenile/Adult onset	Homozygous	[27]
c.610-612delGGA	p.Gly204Δ	Not reported	Heterozygous with Tyr275Cys	[33]
c.622A>T	p.Asn208Tyr	Not reported	Heterozygous with IVS2+1G>A	[7]
c.824A>G	p.Tyr275Cys	Not reported	Heterozygous with Gly204Δ	[33]
c.833C>G	p.Pro278Arg	Not reported	Homozygous	[34]

^a^Numbering starts with A of ATG start codon corresponding to nucleotide 82 of Genbank ID BC103763.1; G>A mutation of guanosine to adenosine; del = deletion

^b^Numbering starts with first methionine of Genbank ID AAI03764; Val183Phe = mutation of threonine 183 to phenylalanine; Gly204Δ = deletion of glycine 204; ins = insertion; aa = amino acid

^c^Mutation of first nucleotide of intron 2; IVS = intervening sequence

## Methods

### Plasmids and site-directed mutagenesis

Plasmids containing myc-tagged eIF2B subunits and His_6_-myc-tagged wild-type (WT) and Val183Phe eIF2Bα have been described previously [2, 8, 22]. Site-directed mutagenesis was carried out using Pfu DNA polymerase (Promega, Southampton, UK) using primers shown in Table 2. The mutations were confirmed by Sanger sequencing of the entire insert.

**Table 2:** Primer sequences used to generate mutant plasmids

Mutation	Mutagenesis primer sequences
Val183Phe	F – 5′ GTG CTA GAT GCT GCT TTC GGC TAC ATC ATG G 3′
	R – 5′ CCA TGA TGT AGC CGA AAG CAG CAT CTA GCA C 3′
Gly204Δ	F – 5′GAA GGA GTT GTT GAA AAC GGA ATT ATT AAC AAG ATT GGA AC 3′
	R – 5′ GTT CCA ATC TTG TTA ATA ATT CCG TTT TCA ACA ACT CCT TC
Asn208Tyr	F – 5′ GAA AAC GGA GGA ATT ATT TAC AAG ATT GGA ACC AAC C 3′
	R – 5′ GGT TGG TTC CAA TCT TGT AAA TAA TTC CTC CGT TTT C 3′
Tyr275Cys	F – 5′ CGT GGG TCG ACT GCA CTG CCC CTT C 3′
	R – 5′ GAA GGG GCA GTG CAG TCG ACC CAC G 3′
Pro278Arg	F – 5′ CGA CTA CAC TGC CCG TTC CTT AAT CAC TC 3′
	R – 5′ GAG TGA TTA AGG AAC GGG CAG TGT AGT CG 3′

### Cell culture, transfection and lysis

HEK293 cells were obtained from ECACC (Salisbury, UK) and maintained in Dulbecco’s modified eagles medium containing 10 % fetal bovine serum and penicillin/streptomycin (Life Technologies, Paisley, UK). Cells were transfected by the calcium phosphate method as described previously [2]. The amount of vector used for each subunit was adjusted to allow equal expression as assessed by western blotting for the myc-tag.

48 h after transfection, lysates of transfected cells were prepared by washing cells twice in cold phosphate-buffered saline (PBS) followed by lysis in 20 mM Hepes-KOH pH 7.6 containing 10 % (v/v) glycerol, 50 mM KCl, 0.5 % Triton X-100, 50 mM β-glycerolphosphate, 14.3 mM β-mercaptoethanol, 0.5 mM EDTA and complete protease inhibitors (Roche). Cell lysates were clarified by centrifugation at 16,000× *g* for 15 min at 4 °C, aliquoted and stored at −80 °C prior to use. A portion of each sample was analysed by western blotting to determine the levels of expression of transfected subunits.

### Analysis of eIF2B complexes

Formation of recombinant eIF2B complexes was carried out as previously described [2, 22]. Briefly, ~200 μg of lysate was applied to 15 μl of Ni^2+^-NTA agarose (Qiagen, Manchester, UK) and topped up to a final volume of 0.5 ml with lysis buffer containing a final concentration of 20 mM imidazole. After mixing for 1 h at 4 °C, the resin was washed 3 times with cold lysis buffer containing 20 mM imidazole and eluted in 30 μl of SDS-PAGE sample buffer containing 250 mM imidazole.

Bound material was analysed by western blot, reprobing the same membrane for myc-tag, total and phospho-eIF2α. Antibodies to phosphorylated (#3597) and total (#2103) eIF2α were purchased from Cell Signalling Technology (Hitchin, Hertfordshire, UK). Anti-myc tag antibody (60003-2-lg) was purchased from Proteintech (Manchester, UK). Anti-β-actin antibody (A4700) was purchased from Sigma Aldrich (Poole, Dorset, UK).

### Measurement of eIF2B nucleotide exchange activity

Measurements of eIF2B GEF activity were carried out as previously described [2, 22]. For analysis of the effects of eIF2 phosphorylation on activity, we phosphorylated purified eIF2 *in vitro*. Briefly, eIF2 was incubated with recombinant PKR (Life Technologies, Paisley, UK) for 30 min at 30 °C in kinase buffer (20 mM Tris–HCl pH 7.5, 10 mM MgCl_2_, 1 mM EGTA, 1 mM Na_3_VO_4_, 5 mM NaF, 2 mM DTT, 0.02 % Triton X-100) containing 200 μM ATP prior to labelling with ^3^H-GDP. Control eIF2 was treated in the same conditions, but excluding PKR and ATP from the buffer.

Data are expressed as mean+/− SEM with the activity of WT complexes being set as 100 %. Statistical analysis was carried out in Graphpad Prism and p-values were generated using an unpaired *t*-test with Welch’s correction.

### Structural rendering

Molecular graphics and analyses were performed with the UCSF Chimera package (v1.9) using the pdf file 3ECS [31, 32]. Chimera is developed by the Resource for Biocomputing, Visualization, and Informatics at the University of California, San Francisco (supported by NIGMS P41-GM103311).

## Results and discussion

### Structural mapping of eIF2B subunits

**Fig. 1:**
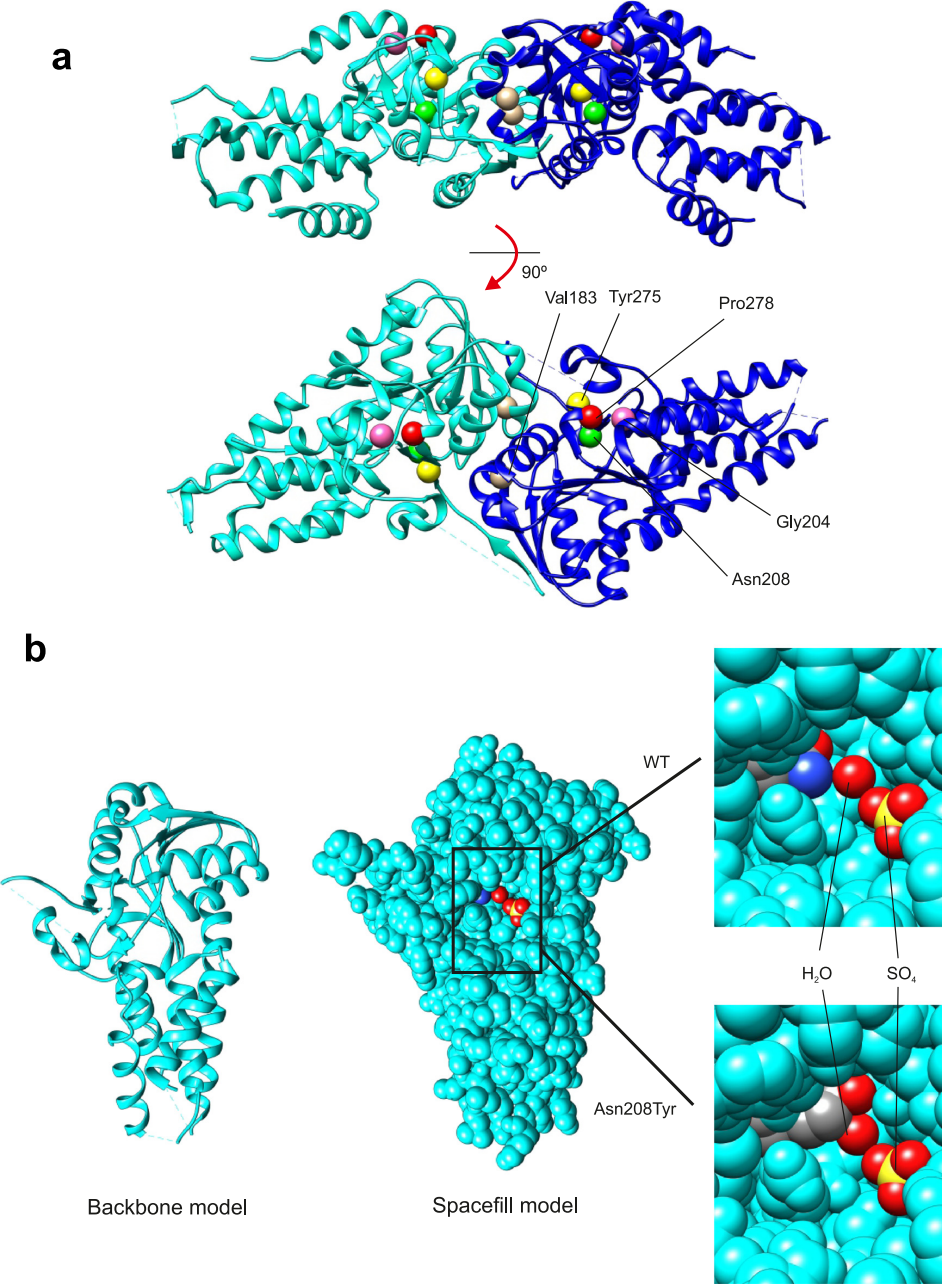
Location of mutations of eIF2Bα on its structure. **a** Structure of the eIF2Bα dimer showing the location of the mutated residues. All the VWM associated mutations affect sites in the α-helix and β-sheet rich Rossmann-like fold rather than the α-helical bundle furthest from the interaction interface of the homodimer. **b** The effect of the Asn208Tyr mutation on the proposed phospho-eIF2 binding pocket. The ribbon and spacefill structures show the location of the pocket on the structure of a single monomer of the subunit. The right hand panels show the WT (Asn208) residue relative to the sulphate ion mimicking a phosphate group and a co-ordinating water molecule (upper panel), and the predicted change to the pocket following *in silico* mutation of Asn208 to Tyr

The atomic resolution structure of eIF2Bα has been previously solved by Hiyama *et al.* [31]. We have recently demonstrated that this subunit forms a homodimer (Fig. 1a) [8]. All the missense or in-frame deletion mutations map to the Rossman-fold domain, comprising two antiparallel β-sheets surrounded by several α-helices (Fig. 1a). The Val183Phe variant maps to the dimeric interface and we have previously shown that this mutation is able to disrupt formation of eIF2Bα homodimers. However, this disruption did not affect formation of eIF2B decamers, thus its effect on eIF2B activity remains unknown [8]. The remaining four sites of mutations, Gly204, Asn208, Tyr275 and Pro278 all occur on two adjacent β-strands in the subunit. Indeed, in the structure, Gly204 and Pro278 are adjacent in space, although they do not appear to interact (Fig. 1a).

Hiyama *et al.* [31] identified a pocket containing a sulphate ion in the structure of eIF2Bα and proposed that, since eIF2Bα is required for the inhibitory binding of phosphorylated eIF2 to the eIF2B complex, this pocket may be the binding site of the phosphorylated serine residue. Interestingly, the side chain of Asn208 protrudes into this site and co-ordinates a water molecule that interacts with the sulphate ion (Fig. 1b). Mutation of this residue to tyrosine may cause a large steric change which may affect binding of phospho-eIF2 into this pocket (Fig. 1b).

### Effects of eIF2Bα VWM mutants on subunit stability and complex formation

**Fig. 2:**
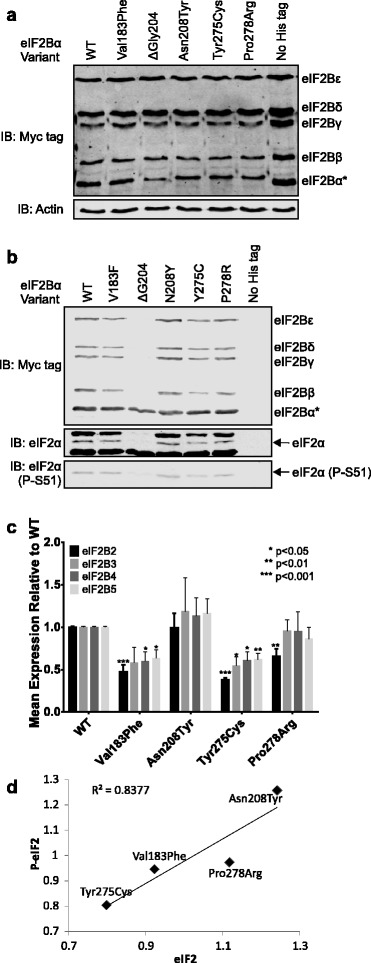
Biochemical effects of mutations of eIF2Bα on eIF2B complex formation. **a** HEK293 cells were transfected with plasmids encoding myc-tagged eIF2B subunits andHis_6_-myc-tagged eIF2Bα. ‘No His-tag’ indicates that a vector encoding myc-tagged eIF2Bα was used instead. Lysates from transfected cells were analysed by western blotting for the myc tag to verify even expression of each subunit. An actin loading control is included. **b** Lysates were subjected to affinity purification using Ni^2+^ agarose and the bound material was analysed by western blot for myc tag to test for the presence of other subunits associated with His_6_-mys-eIF2Bα and phospho- and total eIF2α to assess substrate binding. **c** The myc-signal for the WT and indicated mutations were quantified and normalised to the level of eIF2Bα. Data are shown relative to the level of the WT pulldown. Data are shown as the relative expression to WT ± SEM of 3 independent experiments. *P*-values are as indicated on the figure. **d** Levels of total and phospho-eIF2 from pulldowns were quantified and normalised to the level of eIF2Bα

We over-expressed myc-tagged eIF2B subunits, including hexahistidine (His_6_)-myc-tagged eIF2Bα in HEK293 cells. Since the Asn208Tyr mutation has been previously described as destabilising eIF2Bα when overexpressed in yeast cells [30], we analysed expression of each of the mutant subunits by western blotting (Fig. 2a). We did not find consistent destabilisation caused by any of the mutations, including Asn208Tyr, in contrast to the data of Richardson *et al.* based on studies performed in *Saccharomyces cerevisiae* [30]. This apparent discrepancy is likely due to differences between yeast and mammals.

In order to examine the effects of the mutations on the integrity of eIF2B complexes, overexpressed eIF2B complexes were isolated on Ni^2+^-NTA agarose and analysed by western blot for myc-tag, in order to assess complex formation, eIF2α and phospho-S51-eIF2α, in order to assess substrate binding (Fig. 2b). Quantification of multiple experiments showed that three mutations, Val183Phe, Gly204Δ and Tyr275Cys, affected complex formation (Fig. 2b). The Gly204Δ mutant is unable to interact with any of the other subunits of the eIF2B complex, whereas Tyr275Cys and Val183Phe lead to an approximately 50 % reduction in binding of this subunit to the rest of the complex (Fig. 2b). The other mutations did not affect binding to the other subunits.

A precedent has been set for reduced or loss of eIF2Bα binding to the rest of the eIF2B complex as a means of VWM mutations exhibiting a pathological effect; we have previously shown that the Gly329Val mutation of eIF2Bβ leads to loss of the interaction of eIF2Bα. This is associated with a 50 % reduction of activity of the complexes, identical to the activity of eIF2B complexes entirely lacking eIF2Bα [2]. Thus, loss or reduced binding of eIF2Bα caused by the Gly204Δ and Tyr275Cys mutations, which were reported together in the same patient [33], is likely to result in the disease.

Reprobing the blots for total and phospho-eIF2α revealed no reduction in binding of the substrate to the complexes caused by the different mutants, save where complex formation was affected (Fig. 2b). Quantification of total and phospho-eIF2 binding showed decreased binding of eIF2 to the mutants resulting in decreased complex formation, but no difference in the ratio of eIF2 to phospho-eIF2 was observed (Fig. 2d). This was surprising for Asn208Tyr, given the location of the mutation relative to the predicted phosphate-binding pocket, which would be expected to result in reduced binding to phospho-eIF2 (Fig. 1b). However, it is possible that this pocket is not actually responsible for strengthening the interaction of eIF2B with phosphorylated eIF2α, since the interaction was modelled *in silico* and no experiments have been carried out to confirm this [31].

### Effects of VWM mutations on eIF2B GEF activity

In order to measure the effects of the mutations on eIF2B activity, we carried out GEF activity assays on eIF2B complexes containing WT and mutant eIF2Bα subunits. Since the Val183Phe, Gly204Δ and Tyr275Cys mutations show reduced interaction with the rest of the complex, we would assume that the levels of catalytic ε-subunit would be reduced and any effects on intrinsic activity would thus be hard to interpret, Since eIF2Bβγδε tetrameric complexes can form in the absence of eIF2Bα, reduction or abolition of interaction with other subunits would not lead to total abolition of eIF2B activity, but would instead result in a prevalence of the less active eIF2B(βγδε) tetramers [2, 8]. We have therefore assumed that cells containing these mutations would show reduced eIF2B GEF activity due to a reduction in levels of the most active decameric eIF2B complex. However, we did carry out activity assays on the Val183Phe mutant in order to confirm this reduced activity.

**Fig. 3:**
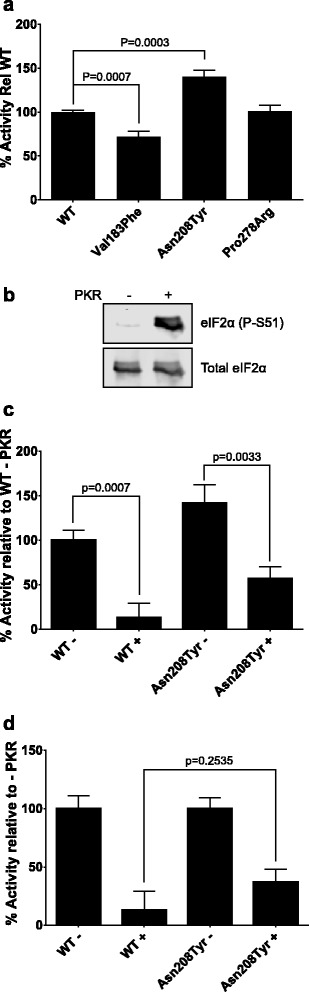
Effects of eIF2Bα mutations on GEF activity and inhibition by phosphorylated eIF2. **a** eIF2B GEF activity assays were carried out on complexes containing the mutant eIF2Bα subunits that do not affect eIF2B complex formation. Data are shown as the GEF activity relative to WT, which is set to 100 %. Activities are shown as mean ± SEM of six independent experiments. *P*-values were calculated by *t*-test with Welch’s correction. **b** Western blot showing the increase in eIF2α phosphorylation following treatment with PKR. **c** eIF2B GEF activity assays were carried out on complexes containing WT or Asn208Tyr eIF2Bα using either unphosphorylated or *in vitro* phosphorylated eIF2. Data are shown as the GEF activity relative to WT, which is set to 100 %. Activities are shown as mean ± SEM of four independent experiments. P-values were calculated by *t*-test with Welch’s correction. **d** The data from (**c**) with the activity for assays with unphosphorylated eIF2 set to 100 % in order to compare the relative decrease in activity

The activity data (Fig. 3a) from multiple experiments show a decrease in GEF activity only for the Val183Phe mutation, which showed an approximately 30 % decrease in activity, as would be expected for the decreased interaction. Intriguingly, the Asn208Tyr mutation causes a significant 40 % increase in GEF activity compared to wild-type complexes. The Pro278Arg mutation has no effect on GEF activity.

The increased GEF activity caused by the Asn208Tyr mutation is not a unique observation among VWM mutations (for example, Val73Gly of eIF2Bε [2]). As described above, the Asn208Tyr mutation was predicted to affect the phosphate binding pocket described in [31]. However, we saw no effect on binding (Fig. 2b). A number of mutations have been described that overcome the inhibitory effects of eIF2α phosphorylation in yeast, in particular the Glu199Lys (Glu198Lys in human eIF2Bα), which also occurs in this predicted pocket [28]. Therefore, it is possible that Asn208Tyr alleviates inhibition by phospho-eIF2, but without affecting its binding, leading to the increase in eIF2B activity. It is unknown how this relates to the disease phenotype. In order to test study this, we carried out *in vitro* GEF assays using eIF2 which is some cases had been pretreated with the eIF2 kinase PKR to phosphorylate it (Fig. 3b). We observed that, as expected, phosphorylation of eIF2 reduced the activity of eIF2B containing either WT or Asn208Tyr eIF2Bα (Fig. 3c). Although complexes containing the Asn208Tyr mutant did tend to show higher activity against phosphorylated eIF2 compared to the wild-type eIF2B (Fig. 3c), this difference was not statistically significant (*p* = 0.2535) (Fig. 3d). Therefore, there is no significant difference in the ability of phospho-eIF2 to inhibit eIF2B containing WT eIF2Bα or the Asn208Tyr mutant.

The lack of an effect of the Pro278Arg mutation on GEF activity is, again, not surprising since a number of mutations, even including some that cause severe disease (such as Ala391Asp in eIF2Bδ) show no effect on either GEF activity or complex formation [2]. It is possible that disruption of, as yet, undescribed functions of eIF2B may underlie the pathological effect of this mutation.

## Conclusions

This study is the first focused study on VWM-associated mutations in the *EIF2B1* gene encoding eIF2Bα. Previous studies have shown effects of VWM mutations on both eIF2B complex formation and GEF activity [2, 22, 23], both of which are observed in this cohort of mutations. However, we also observe that two mutations lead either to increased GEF activity or no change, without affecting complex formation. Along with previous studies, this suggests that these mutations may affect other, as yet undetermined functions of the eIF2B complex. The recently observed GDF activity of the complex sets a precedent for the identification of these alternative functions [26]. Previous studies have also suggested a genotype-phenotype relationship between particular VWM mutations and disease severity [3]. However, a lack of reports on the phenotype for the majority of VWM mutations in *EIF2B1* makes it impossible to identify any such relationships for these mutations. The data in this study add to the increasing body of evidence demonstrating a wide variety of effects of mutations of the eIF2B complex in VWM patients, and shows that we are still some distance from understanding the molecular basis of this disease.

## Abbreviations

eIF: Eukaryotic initiation factor
GEF: Guanine nucleotide exchange factor
SEM: Standard error of the mean
VWM: Vanishing white matter
tRNA: Transfer ribonucleic acid
GDP: Guanosine diphosphate
GTP: Guanosine triphosphate
GDI: GDP dissociation inhibitor
GDF: GDI dissociation factor
